# Single-use cholangioscope-assisted diagnosis of a large sessile serrated lesion within the appendix

**DOI:** 10.1055/a-2512-5473

**Published:** 2025-02-05

**Authors:** Guang Yang, Suhuan Liao, Silin Huang, Jingsong Wang, Jianzhen Ren, Bo Li, Ronggang Zhang

**Affiliations:** 1 Department of Gastroenterology, South China Hospital, Medical School, Shenzhen University, Shenzhen, China

Appendiceal lesions are predominantly discovered incidentally during appendectomy for other indications. Single-use cholangioscopes, used in the management of appendicitis and diverticulitis, facilitate direct visualization of the appendix and diverticula 1 2 . We present a case of a large appendiceal lesion, adeptly diagnosed via direct visualization with a 9-Fr single-use cholangioscope (EyeMax, Micro-Tech, Nanjing, China) ( Video 1 ).

Large sessile serrated lesion within the appendix, diagnosed utilizing a single-use cholangioscope and removed by endoscopic transcecal appendectomy.Video 1

A 55-year-old female patient underwent colonoscopy for chronic constipation and was found to have a whitish lesion encircling the appendiceal orifice, categorized as 0-IIa in the Paris classification and as type 1 in the Japan Narrow-band imaging Expert Team (JNET) classification ( Fig. 1 ). The lesion had a well-defined outer border, extending into the appendiceal lumen. To assess the intraluminal extent of the lesion, a single-use cholangioscope was successfully introduced into the appendiceal lumen ( Fig. 2 ). With water immersion, the lesion manifested as a well-defined, whitish, villiform elevation, encircling the lumen, and proximal to the appendix base. The inner border of the lesion was distinctly demarcated ( Fig. 3 ). Computed tomography revealed a normal appendix. Following consultation with the patient, endoscopic transcecal appendectomy was performed ( Fig. 4 ). Postoperative pathological examination confirmed a sessile serrated lesion (SSL), characterized by distorted serrated crypts, deep crypt serration, and basal crypt dilation ( Fig. 5 ). The patient experienced mild abdominal pain and low grade fever after surgery but recovered quickly with antibiotic treatment and was discharged on the 5th postoperative day.

**Fig. 1:**
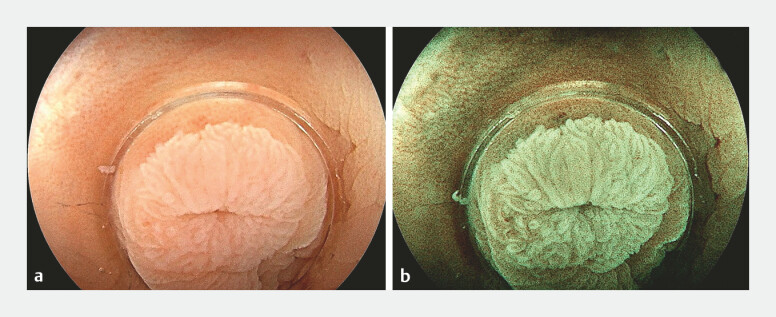
Colonoscopy revealed a whitish lesion encircling the appendiceal orifice, categorized as 0-IIa in the Paris classification and as type 1 in the Japan Narrow-band imaging Expert Team (JNET) classification.

**Fig. 2:**
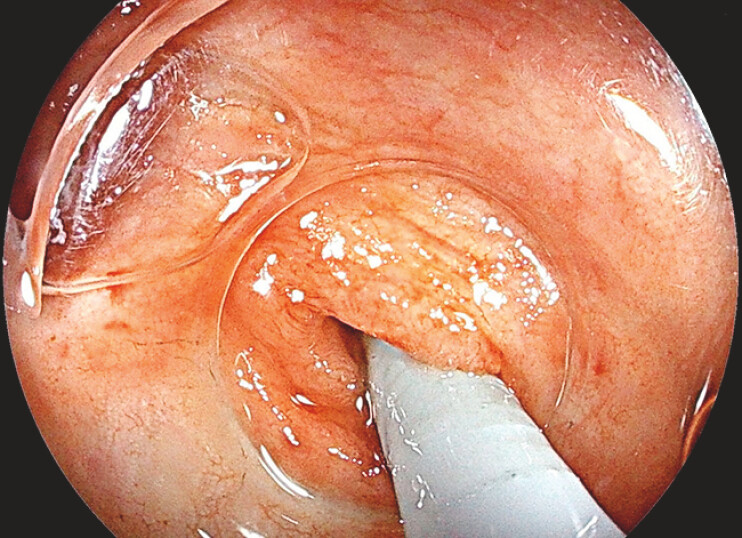
A cholangioscope was inserted into the appendiceal lumen to further assess the intraluminal extent of the lesion.

**Fig. 3:**
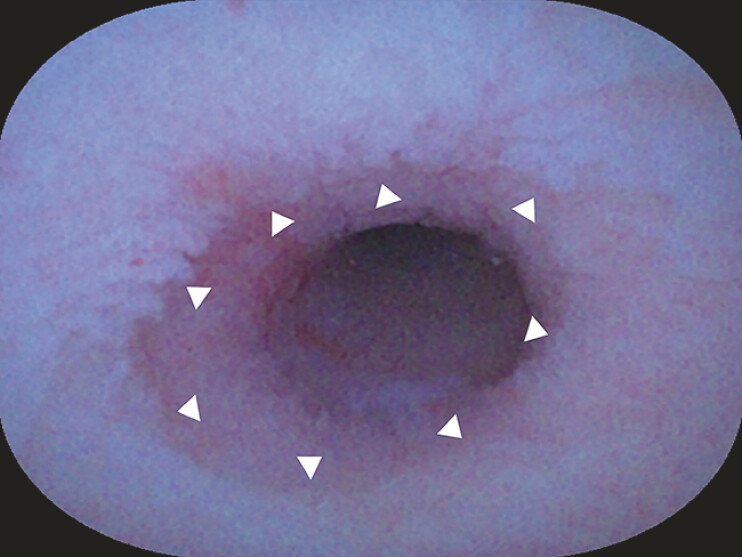
The inner border of the lesion was distinctly demarcated.

**Fig. 4:**
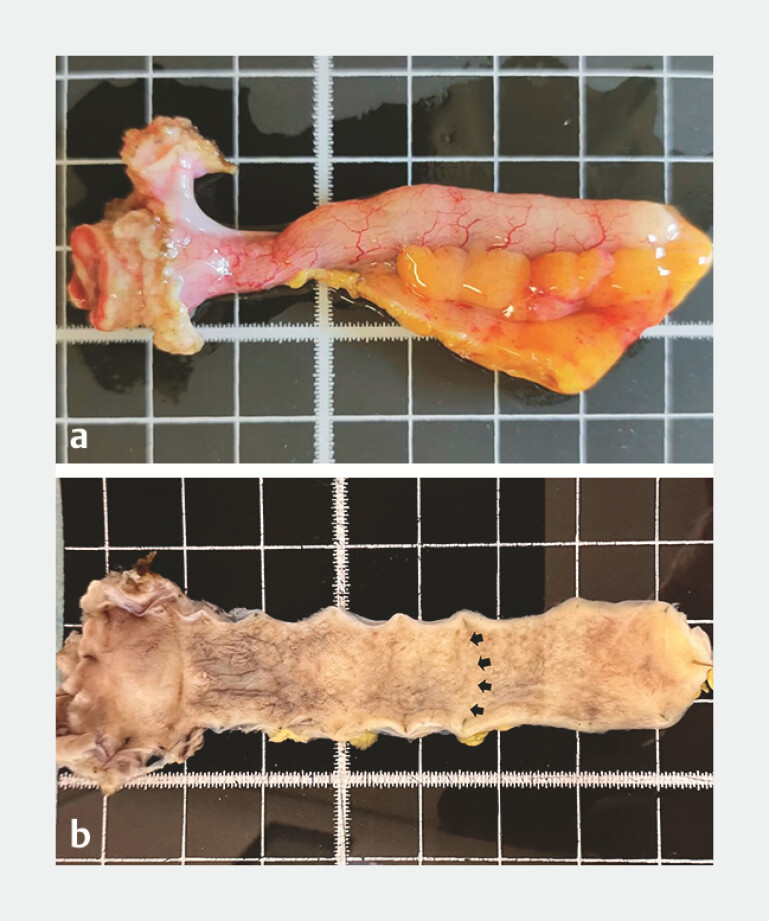
The removed appendix.

**Fig. 5:**
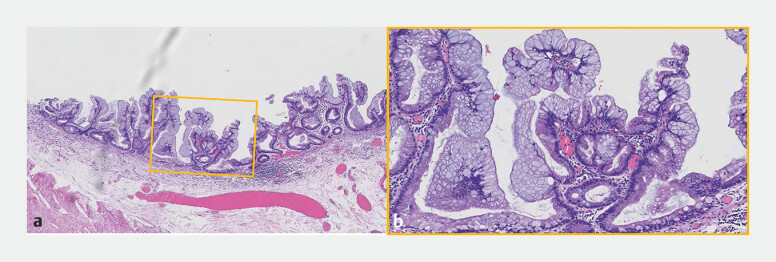
Postoperative pathological examination confirmed a sessile serrated lesion.

SSLs are most commonly found in the right-sided colon and may extend into the appendix 3 . However, diagnosing lesions involving the appendiceal lumen is challenging, as colonoscopy cannot adequately visualize the full extent of these lesions. This case represents the first instance of utilization of a single-use cholangioscope to directly confirm SSL involvement of the appendix, followed by complete endoscopic resection, and offers a valuable reference for the clinical management of similar conditions.

Endoscopy_UCTN_Code_TTT_1AQ_2AD_3AF

## Notes

Endoscopy E-Videos https://eref.thieme.de/e-videos
*E-Videos* is an open access online section of the journal *Endoscopy* , reporting on interesting cases and new techniques in gastroenterological endoscopy. All papers include a high-quality video and are published with a Creative Commons CC-BY license. Endoscopy E-Videos qualify for HINARI discounts and waivers and eligibility is automatically checked during the submission process. We grant 100% waivers to articles whose corresponding authors are based in Group A countries and 50% waivers to those who are based in Group B countries as classified by Research4Life (see: https://www.research4life.org/access/eligibility/ ). This section has its own submission website at https://mc.manuscriptcentral.com/e-videos .
